# A Highly Portable Smartphone-Based Capillary Electrophoresis with Capacitively Coupled Contactless Conductivity Detection

**DOI:** 10.3390/s25072303

**Published:** 2025-04-04

**Authors:** Zhimin Tao, Qiang Zhang, Yiren Cao, Xunjie Duan, Yuyang Wu, Liuyin Fan, Chengxi Cao, Weiwen Liu

**Affiliations:** 1 School of Sensing Science and Engineering, School of Electronic Information and Electrical Engineering, Shanghai Jiao Tong University, Shanghai 200240, China; zhimin.tao@sjtu.edu.cn (Z.T.); billy_zq@sjtu.edu.cn (Q.Z.); caoyiren2010@sjtu.edu.cn (Y.C.); jieaobuxun@sjtu.edu.cn (X.D.); plutochristian@sjtu.edu.cn (Y.W.); 2 Student Innovation Center, Shanghai Jiao Tong University, Shanghai 200240, China; lyfan@sjtu.edu.cn; 3 State Key Laboratory of Microbial Metabolism, School of Life Sciences and Biotechnology, Shanghai Jiao Tong University, Shanghai 200240, China

**Keywords:** capillary electrophoresis, capacitively coupled contactless conductivity detection, metal ions, portability, smartphone

## Abstract

Work has rarely been reported on a highly portable smartphone-based capillary electrophoresis (CE) with capacitively coupled contactless conductivity detection (C^4^D). Herein, a highly portable phone-based CE (130 mm × 190 × 70 mm, 1.4 kg) with C^4^D and Bluetooth communication, as well as user-interface software, was developed for portable analysis. To demonstrate the device, six metal ions were selected as model analytes for verification and successfully applied to the detection of ions in tap water. The analytical performance highlighted that the runs and data analysis of the CE-C^4^D device could be controlled via the user interface based on smartphones. Furthermore, the experiments showed that (i) the linear ranges of six metal ions were between 6 and 1500 μmol/L with a correlation coefficient of more than 0.9934; (ii) the limit of detection (LOD) values were within 1.84–4.33 μmol/L; (iii) the intra-day deviations of migration time and peak area were 2.40–5.24% and 0.75–2.82% (*n* = 5), respectively. Although the LOD is not the most optimal among current portable devices, the results still indicated the satisfactory analytical performance and potential of the developed device, software, and method for portable separation and quantitation of analytes from various fields.

## 1. Introduction

Portable analytical devices contribute to ensuring the living conditions of everyone and enhancing their well-being [1]. They not only eliminate the need for complex sample storage and transportation but also enable on-site assays for prompt decision-making [2,3,4,5,6]. For these reasons, several portable analytical techniques have been developed in the last two decades, such as lateral chromatography [7,8], colloidal gold bioassay [9], electrochemical sensors [10], portable/handheld NIR [11], and Raman spectrophotometers [12,13], as well as electrophoresis titration sensors [14,15].

Capillary electrophoresis (CE) has the advantages of high separation efficiency, short analysis time, low chemical consumption, and easy installation, operation, and maintenance [16], while capacitively coupled contactless conductivity detection (C^4^D) also has the merits of small size, low power consumption, high versatility, and easy construction and operation [17,18,19]. Thus, the combination of CE and C^4^D is particularly suitable for developing portable analytical devices. In 2001, Kappes et al. [20] first proposed a portable CE device with amperometric, potentiometric, and conductometric detection, with dimensions of 340 × 175 × 175 mm and a weight of 7.5 kg. After six years, Kubáň et al. [21] proposed an improved CE-C^4^D device with a size of 310 × 220 × 260 mm and a reduced limit of detection (LOD). Since then, portable CE devices have received increasing attention from research teams. In 2013, Mai et al. [22] designed a portable CE-C^4^D device with a size of 450 × 350 × 150 mm and a weight of 8 kg. One year later, Kobrin et al. [23] and Nguyen et al. [24] designed a CE device and a C^4^D controlled via a computer, respectively. In 2016, Greguš et al. [25] constructed a small CE-C^4^D device with dimensions of 200 × 330 × 170 mm and a weight of less than 5 kg. Koenka et al. [26] presented a thermostatted dual-channel portable CE-C^4^D device with dimensions of 520 × 340 × 180 mm and a weight of less than 15 kg. Opekar et al. [27] designed an original dual-channel CE-C^4^D device on a supporting board. In 2018, Fuiko et al. [28] proposed a CE-C^4^D water quality analyzer integrated in a 19 inch rack. In 2024, Li et al. [29] constructed a compact and high-performance setup of CE-C^4^D with a size of 245 × 220 × 95 mm. Despite the use of LCD tablets or computers for signal acquisition and analysis, the developed CE-C^4^D devices still suffered from unsatisfactory portability for real on-site analysis.

To enhance the portability and practicality of the CE-C^4^D device for field applications, it is essential to focus on miniaturization, operational simplification, and intelligent data processing. Leveraging the ubiquity and user-friendliness of smartphones, these devices can serve as effective platforms for data recording and processing, eliminating the dependency on traditional computers. Notably, smartphones have already demonstrated their potential in advancing bioassay technologies. In 2017, Kanakasabapathy et al. [30] proposed an alternative to blood sample flow cytometry based on smartphones for testing HIV/AIDS. In 2020, Calabretta et al. [31] designed a portable biosensor coupled with paper and smartphone devices for detecting bacterial ATP, Nie et al. [32] demonstrated an electrochemi-luminescence sensor with zinc-doped MoS2 quantum dots (QDs), and Xu et al. [33] developed a smartphone-based point-of-care testing (POCT) system for detection of metal ions. In 2022, Zheng et al. [34] presented a smartphone-based multilayered paper-based microfluidic analysis device for simultaneously determining glucose and uric acid in the blood, and Fiore et al. [35] proposed a smartphone-assisted electrochemical device capable of quantitative analysis of tyrosine in serum samples. In 2023, Yu et al. [36] developed a colorimetric and electrochemical dual-mode smartphone-sensing platform based on MOF-818 nanozyme for in situ detection of hydrogen peroxide and hydrogen sulfide released by living cells. Despite the widespread adoption of smartphones in advancing bioassay technologies, the development of smartphone-based portable CE-C^4^D devices remains unexplored, primarily due to the technical challenges associated with processing complex electrophoretogram data on mobile platforms and achieving seamless integration between CE-C^4^D hardware and smartphone software.

Herein, a highly portable CE-C^4^D device with a smartphone and user interface was designed for real portable analysis. As a proof of concept, six metal ions were selected as model analytes to validate the performance of the developed device. As shown below, the experiments were conducted to systematically validate the device, software and method of CE-C^4^D. Finally, the developed device was successfully used for the actual analysis of drinking water. All of these demonstrate the potential of the developed device, software, and method for portable separation and quantitative analysis of a wide range of analytes in the field.

## 2. CE-C^4^D Device

### 2.1. Design of CE-C^4^D

**Figure 1:**
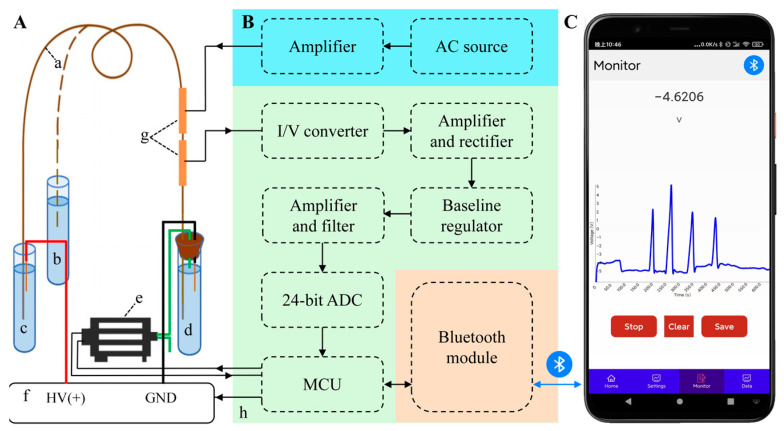
Schematic diagram of the portable smartphone-based CE-C^4^D with three parts of CE separation and cleaning (**A**), the online C^4^D (**B**), and the smartphone (**C**). Panel A: (a) capillary, (b) sample vial, (c) anode BGE vial, (d) cathode BGE vial, (e) micro diaphragm pump, (f) high-voltage power supply, (g) C^4^D-sensing section, and (h) control and signal-processing module.

Figure 1 shows the schematic diagram of the portable smartphone-based CE-C^4^D with three parts of CE separation and cleaning (A), the online C^4^D (B), and the smartphone (C). The first part consists of a capillary (a), a sample vial (b), an anode vial (c) and a cathode vial (d) for background electrolyte (BGE), a micro diaphragm pump (e), and a high-voltage power supply (f), etc. The second part consists of a C^4^D-sensing section with an excitation electrode and a detection electrode (g) and a control and signal processing module (h) with an alternating current (AC) source, an excitation amplifier, an I/V converter, an AC amplifier and rectifier, a baseline regulator, a direct-current (DC) amplifier and filter, a 24-bit analog to digital converter (ADC), a microcontroller unit (MCU), and a Bluetooth module, etc. The last part is the smartphone, which includes an Android phone with the user-interface software for communicating with the CE-C^4^D via Bluetooth.

The CE separation and cleaning part is shown in Figure 1A. Herein, the high voltage electrode and the anode end of the capillary (a) are inserted into the anode BGE vial (c), and when injecting the sample, the anode end of the capillary is inserted from the anode BGE vial into the sample vial (b). The grounding electrode of the power supply and the cathode end of the capillary are inserted into the cathode BGE vial (d) through a rubber plug. In order to achieve automatic injection, the inlet pipe of the diaphragm pump (e) is inserted into the cathode BGE vial through the rubber plug, and the outlet pipe is kept outside. Using a high-voltage power supply (f) with controllable and positive voltage output as the CE power supply.

Figure 1B shows the online C^4^D part. The sensing section (g) is the core component of the C^4^D. Its performance depends not only on its own parameters but also on its position on the capillary. The experiments reveal that the closer the sensing section is to the cathode, the better the ion separation is. The closer the sensing section is to the anode, the faster the ion peak appears. Thus, the sensing section was fixed at 10 cm of the capillary near the cathode based on the preliminary test results. The control and signal-processing module (h) integrates an excitation module, a control and detection module, and a Bluetooth module. The excitation module consists of an AC source and an excitation amplifier. The signal of the AC source is amplified by the excitation amplifier and loaded to the excitation electrode of the sensing section. The control and detection module consists of an I/V converter, an AC amplifier and rectifier, a baseline regulator, a DC amplifier and filter, a 24-bit ADC, and a MCU. The detection electrode converts the received current into a voltage by the I/V converter, amplifies the signal by the amplifier, and finally rectifies it into a DC signal by the rectifier. The DC signal is connected to the DC amplifier and filter after baseline adjustment. The amplified signal is digitized by the 24-bit high-speed ADC and sent to the MCU. The micro diaphragm pump is driven by PWM waves generated by the MCU, and at the same time, feedback pulse signals (6 pulses per cycle) are sent back to the MCU to accurately obtain the pumping volume of the diaphragm pump, thereby improving the accuracy of sample injection. The integrated Bluetooth module is responsible for wireless communication with a smartphone (C) to monitor electrophoresis signal in real-time.

The smartphone is equipped with the user-interface software, which can not only control the run of the CE-C^4^D device, such as controlling the high-voltage power supply and the diaphragm pump, but also receive real-time data from the online C^4^D detector, draw the electrophoretic graphs, and perform data processing, as shown in Figure 1C.

### 2.2. Fabrication of CE-C^4^D Device

**Figure 2:**
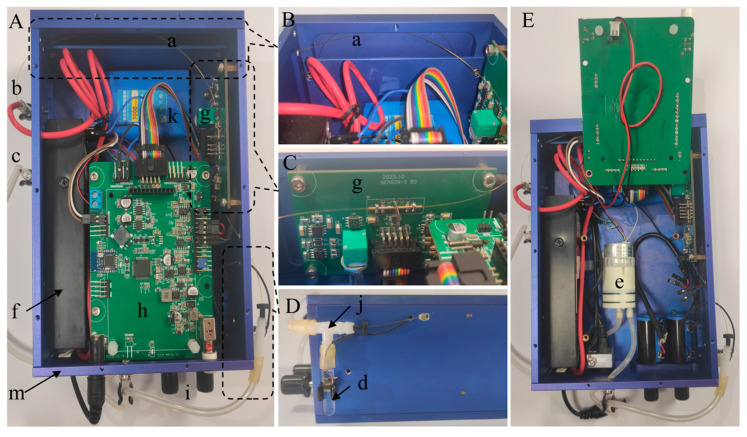
Real photograph of the portable smartphone-based CE-C^4^D device, including top view (**A**), capillary winding diagram (**B**), sensor board diagram (**C**), T-tube diagram (**D**), and internal diagram (**E**). Herein, (a) capillary, (b) sample vial, (c) anode BGE vial, (d) cathode BGE vial, (e) micro diaphragm pump, (f) high-voltage power supply, (g) sensor board, (h) main board, (i) potentiometers for baseline adjustment and amplification, (j) T-tube, (k) lithium battery, and (m) metal shell.

Figure 2 shows the real photograph of the portable smartphone-based CE-C^4^D device, including top view (A), capillary winding diagram (B), sensor board diagram (C), T-tube diagram (D), and internal diagram (E). The developed CE-C^4^D device mainly includes a capillary (a), a sample vial (b), an anode BGE vial (c), a cathode BGE vial (d), a micro diaphragm pump (e), a high-voltage power supply (f), a sensor board (g), a main board (h), two potentiometers for baseline adjustment and amplification (i), a T-tube (j), a lithium battery (k), and a metal shell (m).

The capillary (a) (Ruifeng Chromatograohinc Co., Ltd., Handan, China) with a length of 60 cm (effective length of 50 cm), an outer diameter of 365 μm, and an inner diameter of 75 μm was used. It is threaded through the C^4^D electrode on the sensor board (g), and after spiral winding in the isolation space above the device by relying on the elasticity of the capillary, both ends protrude from the metal shell (m), as shown in Figure 2B,C. One end of the capillary is inserted into the anode BGE vial (c) or the sample vial (b), and the other end is inserted through the rubber plug into one end of the upper outlet of the T-tube (j) and finally extended from the lower outlet of the T-tube and inserted into the cathode BGE vial (d), as shown in Figure 2A,D. Due to the spiral winding of the capillary inside the device, the wound part of the capillary can be pulled out or retracted into the device, thus achieving the purpose of inserting into the anode BGE vial and the sample vial at different positions. Herein, the vials are all Duchenne tubules with a diameter of 6 mm and a length of 30 mm. They are made of glass, can hold approximately 350 μL of solution, and are secured to both sides of the device by adhesive-covered elastic tube clamps. The T-tube (j) is made of polyethylene, and the lower outlet of the T-tube is connected to the cathode BGE vial through a latex sleeve, as shown in Figure 2D. One end of the upper outlet of the T-tube is inserted with the capillary and sealed with the rubber plug, and the other end is connected to the inlet tube of the micro diaphragm pump (e) (H012, Nidec Corporation, Kyoto, Japan) through the latex sleeve to ensure the sealing of the connection part. At the same time, the outlet tube of the diaphragm pump, which operates at a vacuum power of 3 W, is kept outside the device to discharge air, as shown in Figure 2E. When the diaphragm pump is working, it will extract air from the cathode BGE vial, causing the internal pressure to drop below atmospheric pressure, thereby relying on the atmospheric pressure difference between the two ends of the capillary to inject the solution from the anode end into the capillary. Herein, the T-tube and the inlet tube of the diaphragm pump can also serve as air pressure buffers. The high-voltage power supply (f) (KDHM-Q-12S15000P-VI, Xi’an Koso Electronic Technology Co., Ltd., Xi’an, China) has controllable voltage output, with an output voltage range of 0 ~ +15 kV and a volume of only 128 × 40 × 24 mm.

The C^4^D sensor board (g) includes a sensing section, an AC source, and a voltage detection module. It is vertically fixed on the inner side of the device, with the sensing section located about 10 cm away from the cathode end of the capillary. The electrodes of the sensing section are made of copper pipes with an inner diameter of 0.4 mm and an outer diameter of 1 mm. In order to improve the sensitivity of C^4^D, the electrode parameters were selected as a length of 15 mm and a gap of 1 mm according to our previous work [37]. There is a grounded copper plate between the two electrodes as a Faraday shield to eliminate the influence of stray capacitance, as shown in Figure 2C. The AC source used a Wien bridge oscillator base on LF351 to generate a 40 kHz sine wave (Figures S1 and S2), and the excitation amplifier used an operational amplifier AD711 to increase the amplitude of the excitation signal to 10 V. The voltage detection module includes an I/V converter and an amplifier and rectifier. The I/V converter used an operational amplifier OPA637 with a 1 MΩ feedback resistance to convert the picked current into voltage. In addition, another operational amplifier OPA637 is used to further amplify the AC voltage signal. At last, the rectifier was relied on a diode peak detection circuit to convert the voltage signal into a DC signal.

The main board (h) includes a baseline regulator, a DC amplifier and filter, a 24-bit ADC, a MCU, a Bluetooth module, and a power management module. The baseline regulator is obtained via a subtraction circuit based on the OPA227 chip. The DC amplifier and filter are implemented using an active second-order low-pass filtering circuit based on two OPA228 chips, with a signal magnification of 1–500 times. The baseline and magnification can be adjusted by adjusting the potentiometers (i), as shown in Figure 2A. The use of this filter that can both amplify ion peaks and act as a filter has improved the sensitivity of the C^4^D. The ADC uses the ADS1251 to achieve high-precision conversion of analog signal to digital signal. The MCU uses a low-power STM32L053 microprocessor chip with a built-in DA converter for controlling the output of the high-voltage power supply. A low-power Bluetooth module based on TI-CC2541 is used for wireless communication with the smartphone. The device is powered by a 12 V, 3000 mAh lithium battery (k) and can also be connected to an external adapter for charging and power supply. When the adapter is connected, the lithium battery automatically charges. The power management module efficiently distributes power to all components, including the sensor board, main board, and high-voltage module. During high-voltage electrophoresis operation at 15 kV, the device consumes approximately 490 mA of current, enabling the lithium battery to theoretically sustain continuous operation for up to 6 h. Furthermore, the device is housed in an aluminum metal shell (m), with the circuit ground connected to the shell to effectively shield against external signal interference.

### 2.3. Phone-Based User Software

**Figure 3:**
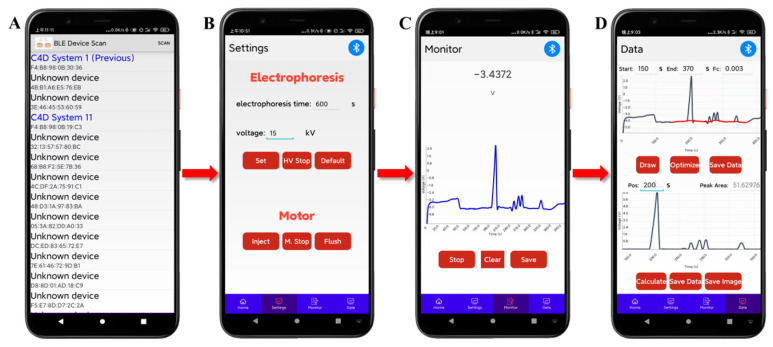
User interface of conductivity detection mobile software for controlling of developed CE-C^4^D device of Bluetooth scanning (**A**), CE condition setting (**B**), CE run (**C**), and data processing (**D**).

A popular smartphone (Xiaomi 6X, Shanghai, China) was used as the user interface. The mobile software is based on Android system, using “activity + fragment + service” components, developed in Java language, and will use the Bluetooth, WiFi, or data traffic function of the mobile phone in the running process. It contains two user interfaces, Bluetooth connectivity and main interface, which also included four sub-interfaces: Home, Settings, Monitor, and Data, as shown in Figure 3. The Settings interface can be used to input or select default electrophoresis voltage and electrophoresis time, control the on/off (“Set”/“HV stop”) of the high voltage, and control the diaphragm pump to complete sample injection and capillary flushing, as shown in Figure 3B. Data visualization in real-time is completed via the Monitor interface, as shown in Figure 3C. The Data interface is developed for data optimization, storage, and calculation, as shown in Figure 3D. Herein, the electropherogram saved in the interface of Monitor can be plotted on the upper display window by clicking the “Draw” button, and then the start and end time of the analysis and the cut-off frequency (Fc) of the filter can be set. By clicking the “Optimize” button, the built-in algorithm can obtain the fitted baseline and the electropherogram after removing baseline and noise within the start and end time, which are plotted on the upper electropherogram and the lower display window, respectively. The data of plotted the fitted baseline can also be saved by clicking the above “Save Data” button. Afterwards, the peak area of the ion peak to be calculated can be obtained by setting the approximate time when the peak appears and clicking the “Calculate” button. If necessary, the data and graph of the electropherogram after removing baseline and noise can be saved in file format on the smartphone by clicking the below “Save Data” button and “Save Image” button, respectively.

The built-in algorithms mainly include the baseline estimation and denoising with sparsity (BEADS) [38] and the Gaussian-peak-fitting algorithm. The BEADS algorithm does not need to carry the distribution characteristics of the baseline, making it more versatile for various electropherograms. It can simultaneously perform baseline estimation and denoising, obtaining baseline and ion peaks. However, the BEADS algorithm can only handle sparse and non-overlapping ion peaks, which cannot handle the overlapping peaks that occur during electrophoresis. The Gaussian-peak-fitting algorithm can accurately separate and reconstruct ion peaks that overlap to a certain extent, making up for the shortcomings of the BEADS algorithm in practical applications. Specifically, the built-in algorithm can automatically identify peaks and valleys based on the derivative of ion peaks and combine with the baseline obtained by the BEADS algorithm to determine whether there are overlapping peaks. If there are overlapping peaks, Gaussian-peak-fitting algorithm is used to separate the overlapping parts and reconstruct them into complete ion peaks. Thus, this algorithm can be used to calculate the area of these peaks and obtain the concentration of the corresponding substances. In addition, due to the insufficient support libraries of Java in fields such as data processing, signal analysis, and numerical computation, the built-in algorithm was implemented on a computer using Python 3.7 and deployed on a cloud server. When processing data, users can set the start and end time of the analysis based on the distribution of the ion peaks to avoid system peaks, set the cut-off frequency of the filter in the BEADS algorithm, and then click the “Optimize” button. The setting parameters and raw data will be uploaded to the cloud server, and the processing results received from the cloud server will be displayed on the smartphone. This process is usually completed within 5 s.

## 3. Materials and Methods

### 3.1. Instruments

An electronic balance (Mettler-Toledo, Greifensee, Switzerland) was used for weighing the sample. The electric blast dryer (Shanghai YIHENG Technical Co., Ltd., Shanghai, China) was used for drying the chemicals. The commercial contact conductivity meter was DDS-307 with DJS-1C conductivity electrode (REX, Shanghai, China). A YD1940A high-voltage digital voltmeter (Changzhou Yangzi Electronics Co., Ltd., Changzhou, China) was used for measuring high voltage.

### 3.2. Chemicals

The chemicals are of analytical grade and can be used without further purification. Standard solutions of the six cations (Mg^2+^, K^+^, Na^+^, Ca^2+^, Zn^2+^, Mn^2+^) were prepared from their sulfates or chlorides. Magnesium chloride was purchased from Bide Pharmatech Co., Ltd. (Shanghai, China). Potassium chloride, sodium chloride, calcium chloride, and zinc chloride were bought from Aladdin Biochemical Technology Co., Ltd. (Shanghai, China). Lactic acid (Lac) was obtained from Titan Technology Co., Ltd. (Shanghai, China). β-Ala was purchased from Sinopharm Chemical Reagent Co., Ltd. (Shanghai, China). All solutions were prepared with deionized water (resistivity of 18.2 MΩ·cm at 25 °C) from SZ-93 automatic distiller (Yarong, Shanghai, China). Tap water was obtained from the laboratory building on campus.

### 3.3. Procedure of CE-C^4^D

First, Lac of 2.815 g and β-Ala of 2.2273 g were weighted, respectively, and were put into a volumetric flask of 1 L to prepare the Lac-β-Ala BGE [39] with a concentration of 25 mmol/L and a pH of 3.6. Store this BGE in a reagent flask for later testing.

Then, stock solutions of 10 mL with concentrations of 4000 μmol/L were prepared for each of the six cations mentioned above. In the experiment, a certain amount of the stock solutions was diluted to 2~1000 folds to obtain sample or mixed-sample solutions with the desired concentrations of each cation for a later test.

Before a run, first, open the developed device and mobile software and connect the phone to the device via Bluetooth. Second, check the rubber plug and latex sleeve to ensure sealing, and click the “Flush” button to flush the capillary. Prior to use, the new capillaries were rinsed sequentially with 0.1 mol/L NaOH, deionized water, and BGE for 10 min each. In the separation process, the capillaries were treated with deionized water and BGE for 2 min each. After rinsing, loosen the rubber plug to balance the air pressure inside and outside the T-tube and then reinsert it. Third, transfer the anode end of the capillary from the anode BGE vial to the sample vial. Fourth, click the “Injection” button to activate the diaphragm pump, which will automatically stop after running for one second. Fifthly, wait for two seconds to achieve the optimal injection time of three seconds (as shown in Figure S3), then release the rubber plug of the T-tube to balance the air pressure and reinsert it to complete the sample injection. Sixthly, move the anode end of the capillary back from the sample vial to the anode BGE vial. Finally, input the required electrophoresis voltage and time in the mobile software settings interface, and click the “Set” button to initiate the CE-C^4^D analysis.

The smartphone can receive the real-time data via Bluetooth and draw electropherogram in the Monitor interface of the mobile software during electrophoresis. At the beginning, users can adjust the baseline to the appropriate position via the baseline potentiometer and wait for the appearance of the ion peaks. After electrophoresis is completed, save the experimental data in the Monitor interface, then perform baseline estimation and denoising and calculate the peak area in the Data interface. If a quantitative calibration curve for a certain ion has been pre-stored, the built-in algorithm can also directly calculate the content of that ion in the sample.

## 4. Results and Discussion

### 4.1. Feasibility of Smartphone-Based CE-C^4^D

**Figure 4:**
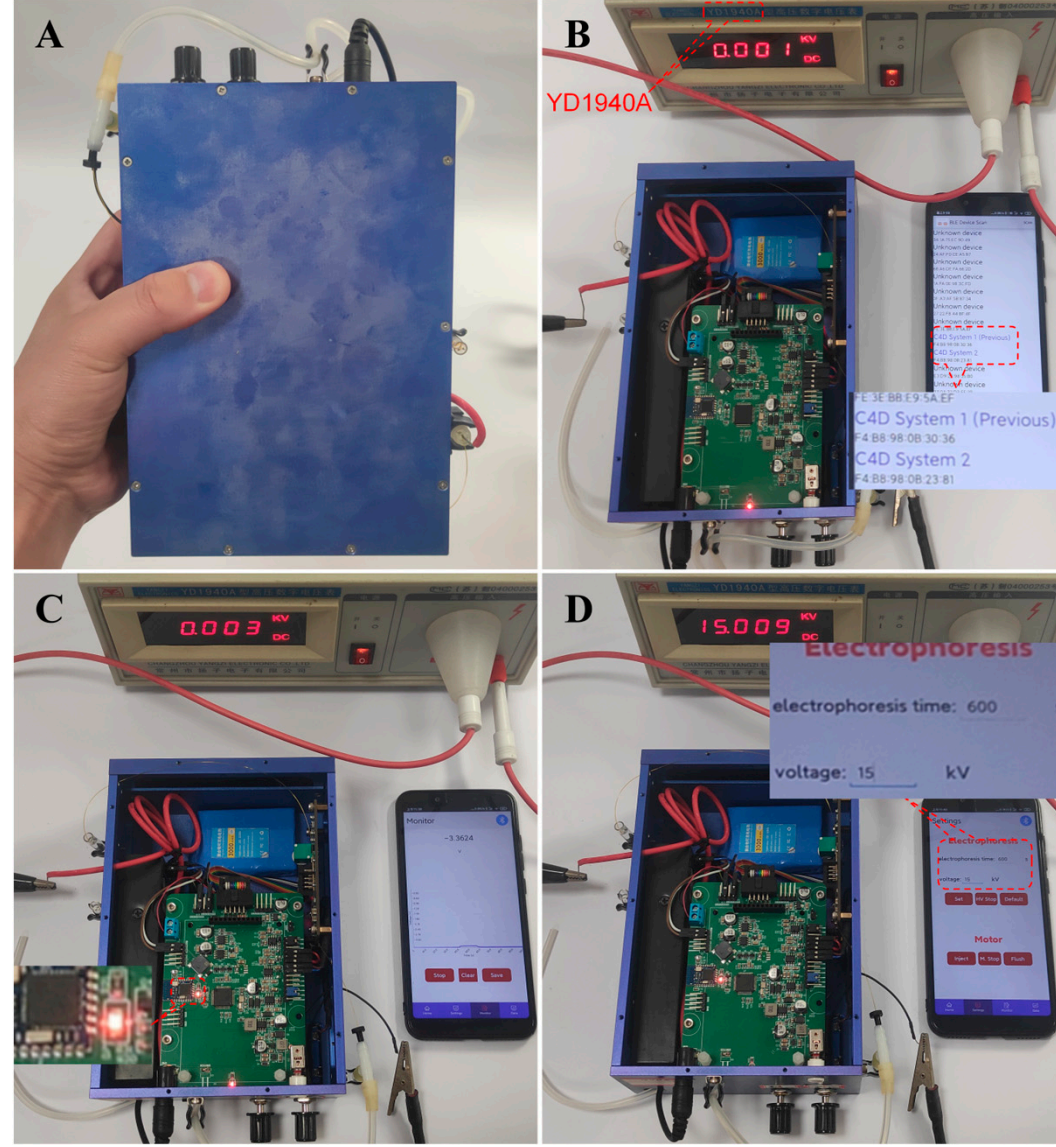
The developed device (**A**) and the core steps, such as Bluetooth scanning (**B**), Bluetooth connecting (**C**), controlling the power supply with the smartphone with a 15 kV output (**D**) of the CE run. Herein, the YD1940A high-voltage digital voltmeter was used to verify the high-voltage output set on the smartphone.

Figure 4 shows the developed device (A) and the core steps, such as Bluetooth scanning (B), Bluetooth connecting (C), and controlling the power supply using a smartphone with a 15 kV output (D) of the CE run. The developed CE-C^4^D device has total dimensions of 130 × 190 × 70 mm (Figure S4), weighs 1.4 kg, and can be held in one hand. At the beginning of the CE run, the smartphone should first search the CE-C^4^D device which was marked with blue name and connected through Bluetooth to the CE-C^4^D device, as shown in panel B. The successfully connected signal on the phone and the indicator light of the device helped the user to make sure the connection was a success (Panel C). Then, after the sample injection, the CE-C^4^D run started, and the real-time data could be visualized on the smartphone, as shown in Figure 3C. After the testing, the user could also use the software developed to conduct the data processing (Figure 3D).

The control of the power supply was performed by the sub-interface of Setting as shown in Figure 4D. A high-voltage meter was used to measure the output of the power supply controlled by the smartphone. The real outputs of the power supply were consistent with the setting voltage, further indicating the feasibility of smartphone-based CE runs.

### 4.2. Analytical Performance

**Figure 5:**
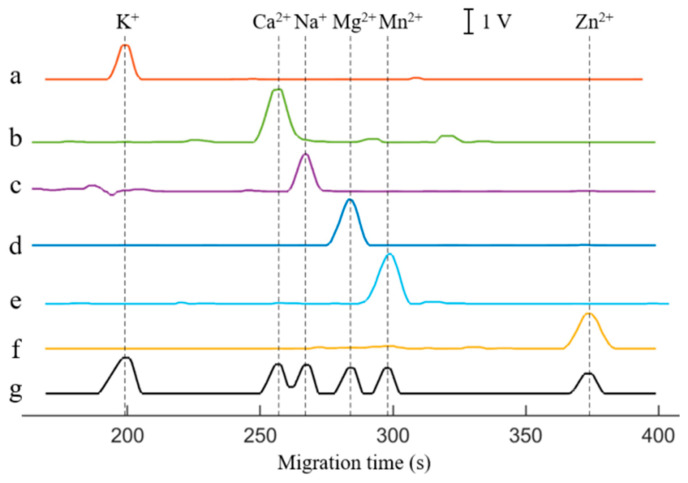
Electrophoretograms for BGE with six single ions and their mixture. a—100 μmol/L K^+^, b—100 μmol/L Ca^2+^, c—100 μmol/L Na^+^, d—100 μmol/L Mg^2+^, e—100 μmol/L Mn^2+^, f—200 μmol/L Zn^2+^, g—mixture. BGE: 25 mmol/L Lac-β-Ala (pH 3.6). Capillary: length of 60 cm (effective length of 50 cm), o.d. of 365 μm and i.d. of 75 μm. Separation voltage: 15 kV. Injection time: 3 s. Excitation source: peak–peak voltage of 20 V and frequency of 40 kHz. DC signal magnification: 100.

Figure 5 presented the electropherograms for BGE with six single cations and their mixtures. The electropherograms of the mixtures showed that K^+^, Mg^2+^, Mn^2+^, and Zn^2+^ could achieve baseline separation within 400 s, with retention times of 199 s, 284 s, 298 s, and 367 s, respectively. It is noteworthy that the reduced ion concentration after mixing enhances electrophoretic separation efficiency, achieving baseline separation of Mg^2+^ and Mn^2+^ in Figure 5g, despite the partial overlap observed in their individual runs (Figure 5d,e). The retention times of Ca^2+^ and Na^+^ were 257 s and 267 s, respectively, but they have not fully achieved baseline separation. Baseline separation of Ca^2+^ and Na^+^ can be achieved by optimizing the separation conditions, such as reducing the capillary inner diameter and simultaneously replacing the BGE (as demonstrated in Figure S5). However, a smaller capillary inner diameter may lead to extended cleaning times and increased susceptibility to clogging. Herein, the Gaussian-peak-fitting algorithm was used to separate and reconstruct ion peaks that overlap to a certain extent, achieving satisfactory results (as shown in Figure S6) and avoiding the aforementioned issues. Overall, the electrophoretogram had a good peak shape, and each peak detected for single metal ion solution could match the peak detected for their mixture. The experimental results fully verified that the CE-C^4^D device had good selectivity for six ions.

Table 1 shows the linear regression equation, coefficients of determination (R^2^), linear range, and LOD of six ions. The results revealed that R^2^ reached 0.9934, and the linear range depended on the species of ions. Herein, the mixture was diluted gradually to obtain the ratio of peak height and noise (S/N) at S/N = 3, and the correspondent concentration of each cation was defined as the LOD for the corresponding cation (Table 1). Accordingly, the LODs for K^+^, Ca^2+^, Na^+^, Mg^2+^, Mn^2+^, and Zn^2+^ were 1.9, 1.8, 3.3, 2.7, 3.6, and 4.3 μmol/L, respectively, implying a good LOD.

Table 2 showed the intra-day and inter-day deviations of the migration time and peak area of the six ions in the smartphone-based CE-C^4^D runs, revealing good stability. Accordingly, the intra-day and inter-day deviations of migration time (MT) of six ions were 2.40–5.24% and 4.17–5.53%, respectively. The intra-day and inter-day deviations of peak area (PA) of six ions were 0.75–2.82% and 1.02–4.15%, respectively.

### 4.3. Application

**Figure 6:**
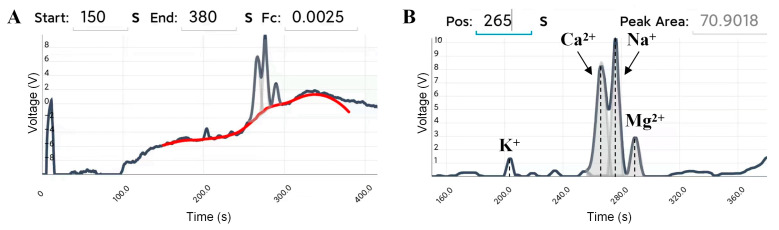
Electrophoretograms for BGE with diluted tap water via the portable smartphone-based CE-C^4^D device, including the baseline fitting (**A**), and peak area calculation (**B**). The dark gray line, red line, and gray line represent the electrophoretic curve, the fitted baseline, and the reconstructed Gaussian peak, respectively. BGE: 25 mmol/L Lac-β-Ala (pH 3.6). Capillary: length of 60 cm (effective length of 50 cm), o.d. of 365 μm and i.d. of 75 μm. Separation voltage: 15 kV. Injection time: 3 s. Excitation source: peak–peak voltage of 20 V and frequency of 40 kHz. DC signal magnification: 100.

The safety of drinking water can be evaluated by detecting the contents of K^+^, Ca^2+^, Na^+^, and Mg^2+^ in tap water. Herein, a certain amount of tap water from the campus was diluted eight times with BGE to prepare a sample solution, and CE-C^4^D experiments were performed according to the previously described procedure. Figure 6 showed the electrophoretograms for BGE with diluted tap water via the portable smartphone-based CE-C^4^D device. The electrophoretogram of tap water showed that the retention times of K^+^, Ca^2+^, Na^+^, and Mg^2+^ were 203 s, 266 s, 276 s, and 290 s, respectively, which were basically the same as the retention times of the corresponding ions in Figure 5.

Table 3 showed the results of eightfold dilution of tap water via the developed CE-C^4^D device and ion chromatography (IC). The CE-C^4^D results showed some deviation from IC measurements, mainly attributed to significant baseline drift in the electrophoretograms and limitations in the data-processing algorithm. Notably, K^+^ exhibited a relatively high recovery rate of 128%, likely influenced by matrix interference and algorithmic inaccuracies. However, the recovery rates of Ca^2+^, Na^+^, and Mg^2+^ ranged between 95% and 106%, demonstrating close agreement with IC results and confirming the reliability of the portable smartphone-based CE-C^4^D device.

### 4.4. Merits and Disadvantages

The developed CE-C^4^D device has the following merits as compared with the existing CE-C^4^D devices (Table 4). First, the developed CE-C^4^D device was of high portability in contrast to the existing CE-C^4^D devices. Its volume was less than 1/3 of the volume of the existed CE-C^4^D portable devices, and its weight was less than 1/4 of the weight of the existing CE-C^4^D portable devices. Second, the developed CE-C^4^D device was controlled by a smartphone in contrast to the existing CE-C^4^D devices. A software developed for control of the smartphone-based CE-C^4^D device facilitates the operation of CE run. Third, the use of the BEADS and Gaussian peak fitting algorithm that can to some extent handle overlapping peaks has improved the applicability of the device. All these made the developed CE-C^4^D device have good portability, facility, and practicability.

While the developed device demonstrates promising performance, its LOD still requires improvement compared to high-performance CE-C^4^D systems [25,29]. Additionally, the migration time repeatability is currently limited by ambient temperature variations and the direct contact between the capillary and the metal shell. Future development efforts should focus on these areas to achieve trace-level detection capabilities and enhanced reproducibility.

## 5. Conclusions

The following conclusions were achieved: First, a highly portable smartphone-based CE-C^4^D device as well as user-interface software was developed for portable analysis. Second, the BEADS algorithm was combined with the Gaussian-peak-fitting algorithm to accurately separate and reconstruct the corresponding partially overlapping ion peaks, avoiding the problems of long capillary cleaning time and easy blockage caused by using smaller inner-diameter capillaries. Third, the experiments of the CE-C^4^D device were systemically performed, which well demonstrated the validity of the device, software, and method of CE-C^4^D. Finally, the developed CE-C^4^D device was successfully applied to the real analysis of tap water, and the results were basically consistent with the IC. The developed CE-C^4^D device had the following merits of good linearity, low LOD, good repetition, and high accuracy. Moreover, the developed CE-C^4^D device has better portability in contrast to the existed CE-C^4^D portable devices. All these indicate that the developed device, software, and method have the potential for portable separation and quantification of analytes in various fields.

## Figures and Tables

**Table 1:** Linear regression equation, coefficients of determination, linear range, and LOD of six ions.

Ions	Regression Equation	R^2^	Linear Range (μmol/L)	LOD (μmol/L)
K^+^	y = 0.1958 × x + 1.5112	0.9995	6–800	1.9
Ca^2+^	y = 0.3742 × x + 21.602	0.9934	6–1000	1.8
Na^+^	y = 0.2081 × x + 4.4588	0.9953	10–1000	3.3
Mg^2+^	y = 0.3261 × x + 4.7685	0.9989	10–1000	2.7
Mn^2+^	y = 0.1868 × x + 5.5635	0.9990	12–1500	3.6
Zn^2+^	y = 0.1845 × x + 0.6490	0.9982	15–1500	4.3

**Table 2:** Intra-day and inter-day deviations of migration time and peak area of six ions in the phone-based CE-C^4^D runs.

Ions	RSD of Intra-Day (%) (*n* = 5)		RSD of Inter-Day (%) (*n* = 5)	
	Migration Time	Area	Migration Time	Area
K^+^	2.40	2.53	4.68	3.34
Ca^2+^	5.24	1.38	5.53	1.87
Na^+^	4.96	0.75	5.34	1.02
Mg^2+^	3.05	2.03	4.52	2.69
Mn^2+^	3.83	2.70	5.22	4.15
Zn^2+^	3.54	2.82	4.17	3.03

**Table 3:** Results of eightfold dilution of tap water via the developed CE-C^4^D device and IC.

Ions	Developed CE-C^4^D		IC (Table S1)	Recovery
	Peak Area	Detected (μmol/L)	Detected (μmol/L)	
K^+^	6.1634	23.76	18.53	128%
Ca^2+^	70.9018	131.75	127.30	103%
Na^+^	57.9601	257.09	241.41	106%
Mg^2+^	19.8852	46.36	48.76	95%

**Table 4:** Comparison of developed CE-C^4^D device with existing pCE-C^4^D devices.

Devices	Size (mm^3^)	Weight (kg)	Phone Based	LOD (μmol/L)	RSD (MT)	RSD (PA)	Recovery (%)	Analytes	Ref.
CE-C^4^D prototype	340 × 175 × 175	7.5	No	0.7–5.6	-	-	-	Inorganic ions, organic acids	[20]
New portable CE-C^4^D	310 × 220 × 260	-	No	0.2–1.0	-	0.82–6.9	-	Inorganic cations and anions	[21]
Suitcase CE-C^4^D	450 × 350 × 150	8	No	1.5–17.0		0.6–2.4	93–116	Inorganic cations and anions	[22]
Purpose-built CE-C^4^D	270 × 246 × 124	-	No	3.7–35.7	0.3–0.6	3.6–9.9	-	Inorganic cations and anions	[23]
semi-auto CE-C^4^D	400 × 280 × 210	6	No	2.1–3.4 ^a^	0.8–0.9	4.1–6.2	86.6–113.9	β-agonists	[24]
Portable CE-C^4^D	200 × 330 × 170	<5	No	0.04–0.36	0.42–1.06	2.85–5.05	-	Inorganic and organic anions	[25]
Dual channel CE	520 × 340 × 180	<15	No	2.8–18	-	6.3–11.6	-	Inorganic cations and anions	[26]
On-board CE	200 × 250 ^b^	-	No	6.9–10.6	1.5–3.5	2.0–5.2	95.2–114.5	Inorganic cations and anions	[27]
SIA-CE-C^4^D ^c^	19 inch rack	-	No	0.5–3.4	0.43–0.96	0.52–1.34	90–108	NO_2_^−^, NO_3_^−^, NH_4_^+^	[28]
Compact CE-C^4^D	245 × 220 × 95	-	No	0.029–0.41	-	-	97.1–103.7	Lanthanide rare-earth ions	[29]
Phone-based CE-C^4^D	130 × 190 × 70	1.4	Yes	1.8–4.3	2.40–5.53	0.75–4.15	95–106(K^+^:128)	Inorganic cations	This work

^a^ The LOD of this was transformed from the determination of β-agonists with a unit of ppm. ^b^ the device was set on a 200 × 250 board and the depth was not mentioned. ^c^ SIA was the brief name of sequential injection analysis.

## Data Availability

The original contributions presented in this study are included in the article/Supplementary Material. Further inquiries can be directed to the corresponding author(s).

## Supplementary Materials

The supporting information can be downloaded at https://www.mdpi.com/article/10.3390/s25072303/s1, Figure S1: Schematic diagram of the C^4^D; Figure S2: Output and sensitivity characteristic curves of the C^4^D with different excitation frequencies; Figure S3: Electrophoretograms with different injection times; Figure S4: Left- and right-side photograph of the CE-C^4^D device; Figure S5: Electrophoretogram for BGE with mixed solution of six ions; Figure S6: The accuracy of fitting-ion peaks with different degrees of overlap; Table S1: Ion chromatography results of tap water.
