# Supplementary material for: A Highly Portable Smartphone-Based Capillary Electrophoresis with Capacitively Coupled Contactless Conductivity Detection
Source: Sensors (Basel). 2025 Apr 4;25(7):2303. doi: 10.3390/s25072303 (PMC11991536; doi:10.3390/s25072303)
Supplement: Supplementary file 1 [file sensors-25-02303-s001.zip › sensors-3541576-supplementary.pdf]

Supplemental Material

A Highly Portable Smartphone-Based Capillary Electrophoresis with Capacitively Coupled Contactless Conductivity Detection

Zhimin Tao, Qiang Zhang, Yiren Cao, Xunjie Duan, Yuyang Wu, Liuyin Fan, Chengxi Cao and Weiwen Liu

Abbreviations: CE, capillary electrophoresis; **C^4D**, capacitively coupled contactless conductivity detection; **CE-C^4D**, capillary electrophoresis with capacitively coupled contactless conductivity detection.

S1. The parameters optimization and analysis of the sensing unit of C^4D

Figure S1 shows the schematic diagram of the C^4D. **Figure S1A** presents the construction of the C^4D, including an insulating pipe, an excitation electrode, a detection electrode, a Faraday shield, an alternating current (AC) source, and an I/V converter. The equivalent circuit is simplified (**Figure S1B**) for analyzing the principle of the C^4D. The two electrodes and the electrolyte solution form the two coupled-wall capacitances (C_w) through the insulating pipe, and the solution between the two electrodes is equivalent to a resistor of R. Due to the existence of the Faraday shield, a leakage capacitance (C_L) is formed between the electrolyte solution and the Faraday shield, while the level of stray capacitance (C_S) between the two electrodes is greatly reduced, according to simplified equivalent circuit:

$$\begin{cases} V_i = \dfrac{1}{j\omega C_s} \times I_1 \\ V_i = (\dfrac{R}{2} + \dfrac{1}{j\omega 2C_w}) \times I_2 + (\dfrac{R}{2} + \dfrac{1}{j\omega 2C_w}) \times I_3 \\ V_i = (\dfrac{R}{2} + \dfrac{1}{j\omega 2C_w}) \times I_2 + \dfrac{1}{j\omega C_L} \times I_4 \\ I_2 = I_3 + I_4 \end{cases} \quad \text{(S1)}$$

The output of the C4D is $V_o = (I_1 + I_3) \times \frac{R_f}{1+j\omega R_f C_f}$, which is expressed as

$$V_o = V_{pp} \times (j\omega C_s + \frac{1}{R + \dfrac{1}{j\omega C_w} + \dfrac{RC_L}{2C_w} + \dfrac{1}{4}R^2 j\omega C_L + \dfrac{C_L}{j\omega 4C_w^2}}) \times \frac{R_f}{1 + j\omega R_f C_f} \quad \text{(S2)}$$

where j is an imaginary unit, ω is the angular frequency of AC source, and V_{pp} is the peak-to-peak value of AC

source. Specifically, $R=\frac{4(l+d)}{\sigma\pi D_{i2}^2}$, $C_W=\frac{\pi\varepsilon_0\varepsilon_r}{\ln(D_{i1}/D_{o2})+\varepsilon_r\ln(D_{o2}/D_{i2})}$, $C_S=\frac{\pi\varepsilon_0(D_{o1}-D_{o2})^2}{\ln(D_{i1}/D_{o2})+\varepsilon_r\ln(D_{o2}/D_{i2})}$; ε_0 is the absolute permittivity, ε_r is the relative permittivity of the insulating pipe, l is the length of the electrode, d is the gap of excitation and detection electrodes, σ is the conductivity of the solution, D_{i1} and D_{o1} are the inner diameter and the outer diameter of the electrodes, and D_{i2} and D_{o2} are the inner diameter and the outer diameter of the insulating pipe, respectively. Based on eq. (S2), the sensitivity of the sensor can be expressed as follows:

$$S=\frac{\Delta V_o}{\Delta\sigma} \tag{S3}$$

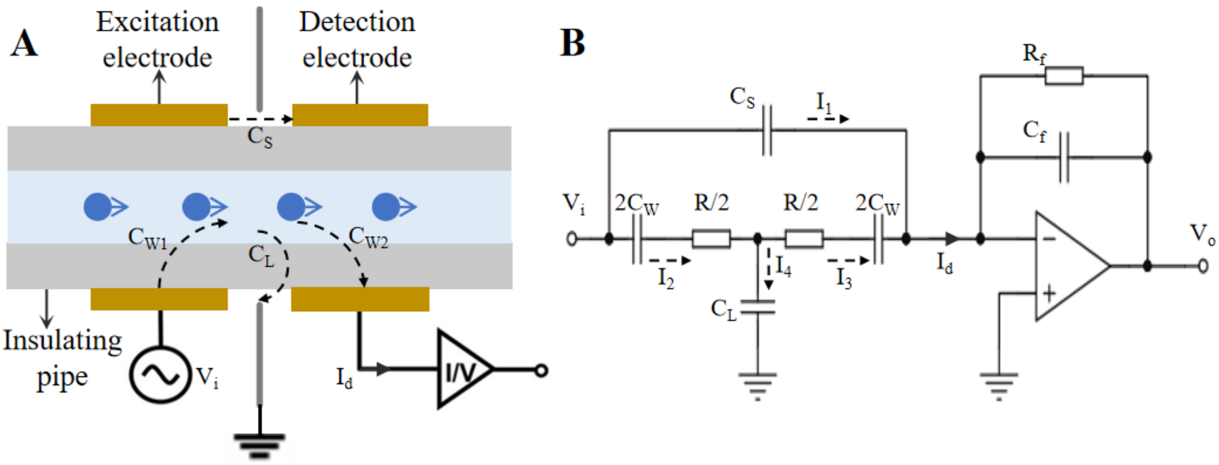

Figure S1. Schematic diagram of the C^{4}D. (**A**) Construction of the C^{4}D and (**B**) its simplified equivalent circuit.

A further digital computation about the sensing unit of C^{4}D was performed for obtaining a more suitable sensitivity at a low conductivity range, and the output and sensitivity characteristic curves of the C^{4}D were obtained according to Eq. (S2) and (S3). Herein, the excitation frequency was considered. **Figure S2** shows that when the frequency of the electrodes was lower, the output of the C^{4}D was higher in the range of low conductivity, as shown in **Panel A**. Meanwhile, a length of 10 mm might have a great sensitivity in the range of low frequency, but it had extremely low sensitivity in the high conductivity range, as shown in **Panel B**. The experimental results are basically consistent with the simulation results, with only a slight difference in the cross position of the sensitivity curves. Taking into account that the conductivity of the background solution is approximately 400 µS/cm, we ultimately selected the parameters of the sensing unit with an excitation frequency of 40 kHz for the self-made C^{4}D module.

S2. Excitation frequency optimization simulation and experiments

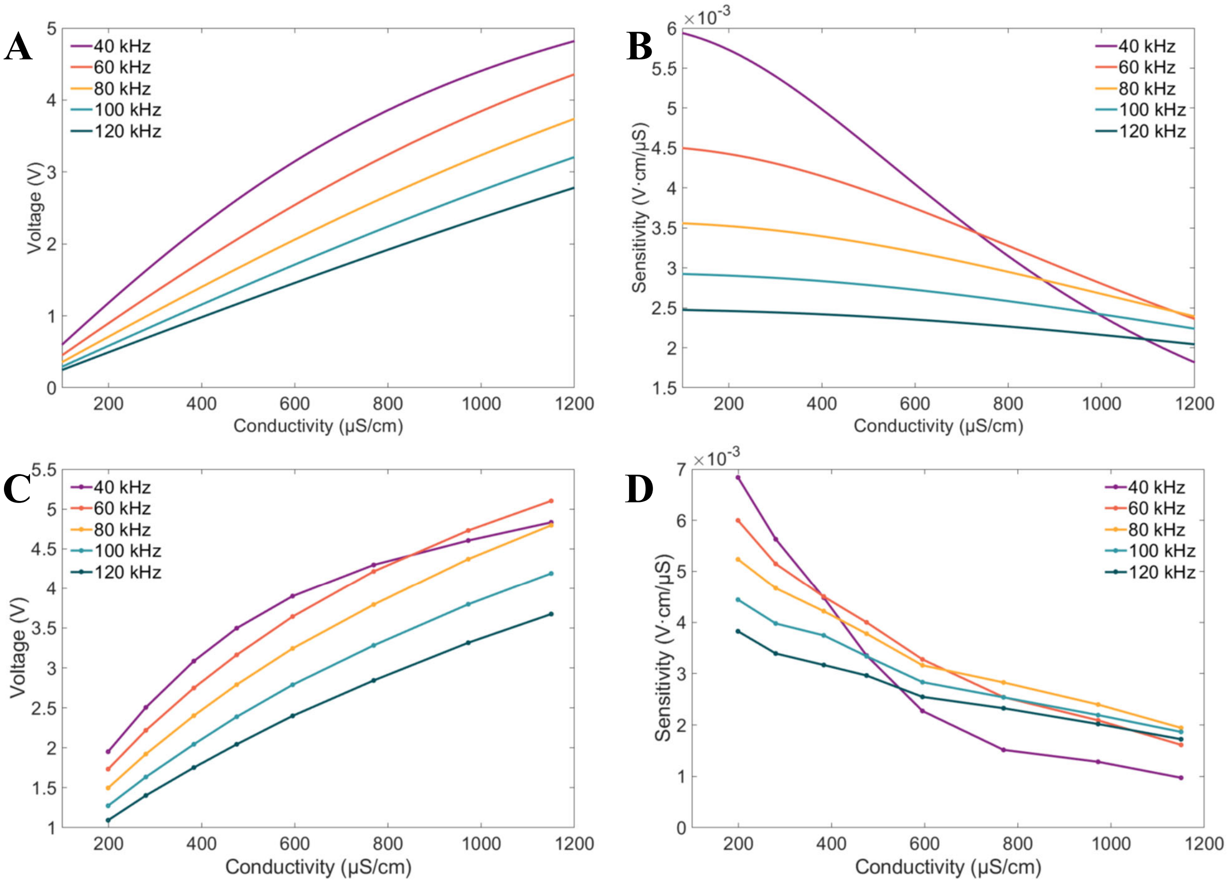

Figure S2. Output and sensitivity characteristic curves of the C^4D with different excitation frequencies: (**A**) simulation output, (**B**) simulation sensitivity, (**C**) experimental signal output, and (**D**) experimental signal sensitivity. $l = 10$ µm, d = 1mm, D_{i1} = 400 µm, D_{o1} = 500µm, D_{i2} = 75 µm, V_{pp} = 20 V, R_f = 1 MΩ, C_f = 5 pF, C_L = 1 fF.

S3. Electrophoretograms with different injection times

Figure S3 shows the electrophoretograms of the same sample at different injection times. Possibly due to internal capillary attachment, the first peak of all the profiles showed a trailing front. In contrast, the electrophoretic peaks of 1s sampling and 3s sampling are more consistent with Gaussian peaks, and the baseline is more stable. In contrast, the baseline fluctuation was more obvious in the electrophoretograms with 5 s sampling. As a result, the 3 s injection time was chosen based on a balance between obtaining a sufficient amount of sample for accurate detection and minimizing potential issues, such as sample overloading and band broadening.

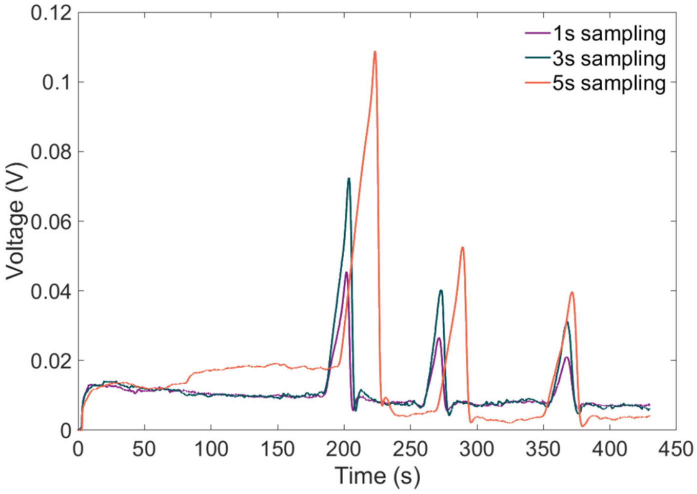

Figure. S3 Electrophoretograms with different injection times. Sample: 300 μmol/L K^+, 20 μmol/L Ca^{2+}, 100 μmol/L Zn^{2+}. BGE: 25 mmol/L Lac-β-Ala (pH 3.6). Capillary: length of 60 cm (effective length of 50 cm), o.d. of 365 μm and i.d. of 25 μm. Separation voltage: 15 kV. Injection time: 1 s, 3s and 5s. Excitation source: peak-peak voltage of 20 V and frequency of 40 kHz. DC signal magnification: 1.

S4. Photograph of the CE-C4D device

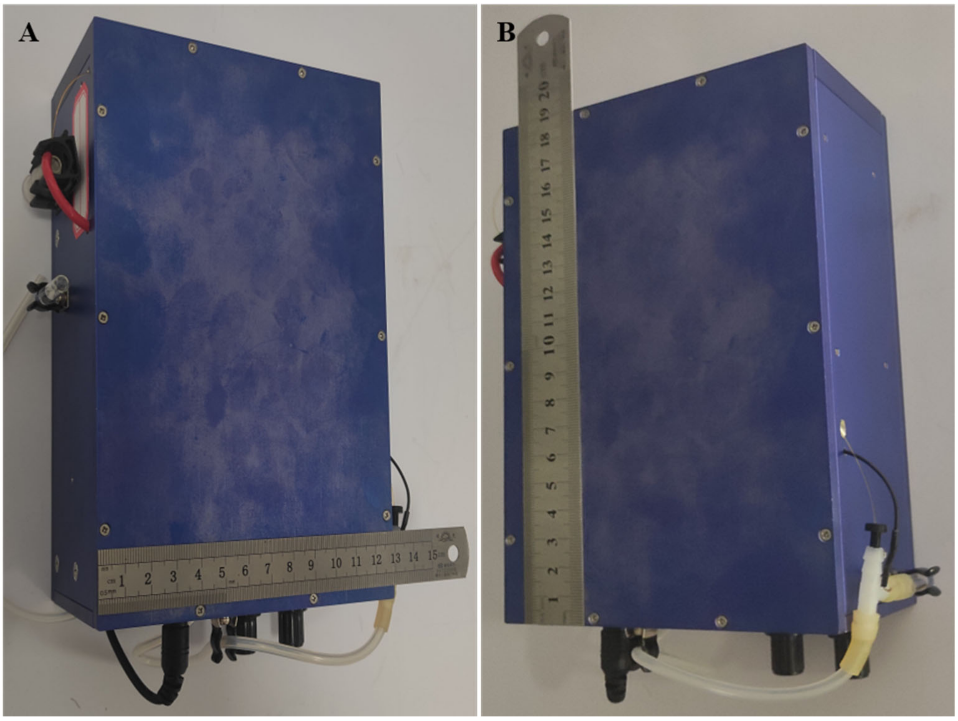

Figure. S4 Left- (**A**) and right (**B**)-side photograph of the CE-C4D device.

S5. Electrophoretogram of six ions under other separation conditions

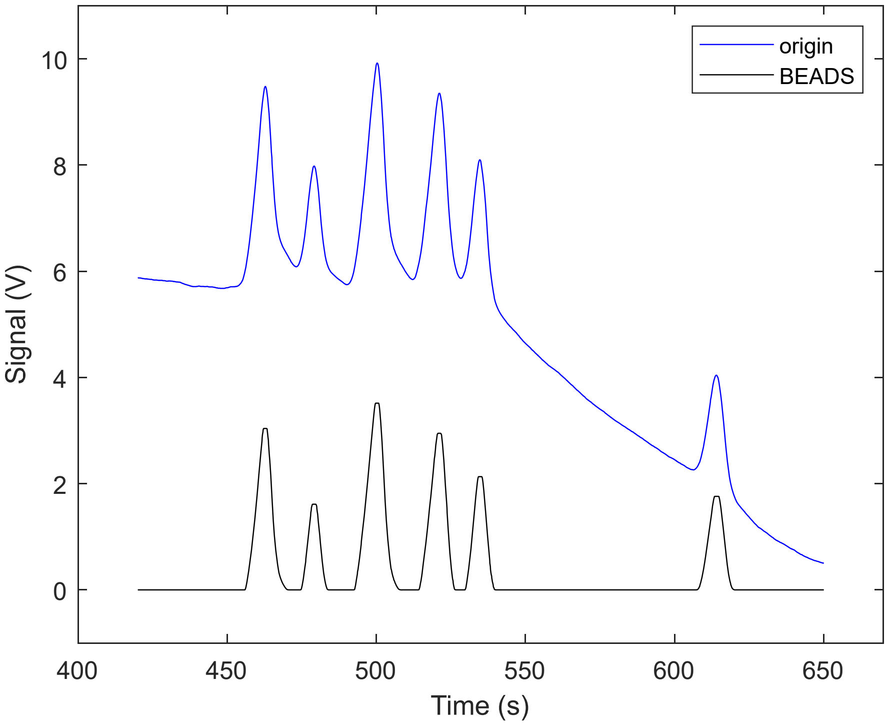

Figure. S5 Electrophoretogram for BGE with mixed solution of six ions. Sample: 2.5 mmol/L K^+, Ca^{2+}, Na^+, Mg^{2+}, Mn^{2+}, Zn^{2+}. BGE: 9 mmol/L His, 4.6 mmol/L Lac, 25 mmol/L HAC, 1 mmol/L 18-crown-6 (pH 3.8). Capillary: length of 60 cm (effective length of 50 cm), o.d. of 365 μm and i.d. of 25 μm. Separation voltage: 15 kV. Injection time: 3 s. Excitation source: peak–peak voltage of 20 V and frequency of 40 kHz. DC signal magnification: 100.

S6. The accuracy of fitting peaks

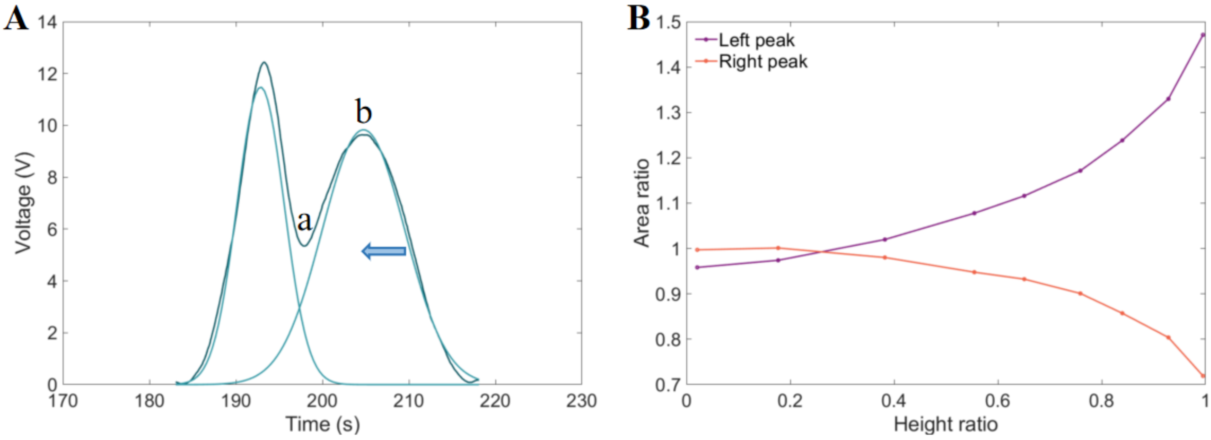

Figure. S6 The accuracy of fitting-ion peaks with different degrees of overlap.

Figure S6 shows the results of the experiments on the accuracy of fitting-ion peaks with different degrees of overlap. Consider two real CE ion peaks that approximate Gaussian peaks. At first, the two ion peaks are essentially non-overlapping, at which time the ion peaks have an area of S_{true}. With the left ion peak kept stationary and the right ion peak moved to the left, as the movement proceeds, the two ion peaks begin to overlap, and the overlapped peak shape contains the trough **a**, as shown in **Figure S6A**. The overlapped peak shapes are reconstructed by the algorithm, the area S_{fit} of the fitted Gaussian peaks is calculated separately, and the fitting accuracy is expressed as the area ratio, which is S_{fit}/S_{true}. As the overlapped portion increases, the height H_a at point a of the trough increases, and the height H_b at point b remains constant. The overlap of the two ion peaks is expressed as the height ratio, which is H_a/H_b. The relationship between the degree of overlap and the fitting accuracy is shown in **Figure S6B**. Since the ion peaks themselves are not fully Gaussian, the fitting accuracy is not exactly 1 at the beginning. When the overlap is less than 0.6, the fitting accuracy of both ion peaks is greater than 90%. When the overlap reaches 0.75, the right ion peak is still fitted with 90% accuracy. The experimental results show that the Gaussian-peak-fitting algorithm is able to solve the overlapping problem of two ion peaks within a certain degree.

S7. Detection results of tap water

Tap water was taken from the laboratory building on campus, sealed, and partially sent to the Testing and Analysis Center of Shanghai Jiao Tong University for quantitative testing. The instrument used for detection was a Dionex ICS-5000+ ion chromatography system. The detection results were shown in **Table S1**.

Table S1. Ion chromatography results of tap water

ions	mass concentration (mg/L)	molar concentration (μmol/L)
K^+	5.80	148.22
Ca^{2+}	40.82	1018.39
Na^+	44.40	1931.29
Mg^{2+}	9.48	390.04
Li^+	not detected	not detected
NH_4^+	not detected	not detected
